# Free spreader grafts in rhinoplasty

**DOI:** 10.1007/s00238-015-1085-2

**Published:** 2015-05-08

**Authors:** Yves Goffart, Sarah Karelle, Jacques Daele

**Affiliations:** Department of ENT/Head & Neck Surgery, CHR Citadelle, Bvd XII de Ligne, 4000 Liège, Belgium

**Keywords:** Rhinoplasty, Spreader grafts, Surgical technique, Cartilage grafting, Middle vault, Fibrin glue

## Abstract

**Background:**

Spreader grafts (SPG) are widely used for different purposes in rhinoplasty procedures. However, selection of the size of the grafts, trimming and fixation often proved time consuming and difficult. We used an original method of placement of “free” SPG to improve both ease of placement and fine trimming of the grafts. To assess pertinence of this approach, we evaluated retrospectively our rate of correction of the middle third of the nose.

**Methods:**

We used a personal method for securing SPG after suturing upper lateral cartilages (ULC). Grafts were inserted between the ULC and nasal septum, adding fibrin glue for fixation. We reviewed the aesthetic results of a series of 420 consecutive rhinoplasties in whom free SPG were used in 218 patients and conventional fixed SPG were used in 33 patients. Retrospective analysis of the photographic data of all patients was performed. Adequacy of brow tip lines, symmetry and relative width of the middle third was assessed by an independent observer and the surgeon.

**Results:**

No evidence of postoperative displacement was noted. Symmetry of the middle third of the nose and adequacy of aesthetic brow tip lines were obtained in the vast majority of the patients. Comparable rates of middle vault correction and harmony were obtained in patients with free SPG or conventional SPG. On occasion during revision surgeries, we found the grafts resting in adequate position.

**Conclusions:**

Free SPG placement appeared a straightforward and timesaving method in rhinoplasty procedures and proved especially meaningful when limited to moderate amount of support was needed in the middle vault reconstruction. Repositioning, resizing of the graft or placement of additional pieces of cartilage were feasible instantly if needed. This technique might help to obtain better correction of the middle third due to easiness and possibility of fine adjustments in rhinoplasty procedures.

Level of Evidence: Level III, therapeutic study.

## Introduction

Sheen first described spreader grafts (SPG) to reconstruct the middle vault after dorsal reduction, and this technique proved one of the most valuable graft in rhinoplasty [1, 2]. The initial Sheen technique was performed endonasally by placement of SPG into submucous tunnels. Variations in the technique have been described, such as endonasal placement with loop sutures [3], with spreader flap by folding the upper lateral cartilages (ULC) [4], or by various shapes of the grafts [5, 6], although the advent of open rhinoplasty procedures contributed extensively to the development of SPG [7].

SPG are used for different and frequent purposes: avoiding inferomedial collapse of the ULC after dorsal reduction, maintaining dorsal aesthetic lines after osteotomies, correcting asymmetries of the middle third and repairing avulsion of the ULC. Their role can also be functional when opening the nasal valve is needed or structural when used for straightening a deviated dorsal septum or lengthening a short nose [8].

SPG are made of autologous cartilage and are sutured alongside the nasal septum. These grafts may stand level with the dorsal septum or slightly lower if adequate height of ULC is present. There are no standard sizes but dimensions of 15–25 mm length, 1–2 mm width are often reported. They tend to be larger in revision surgery, longer in extended SPG. A careful trimming of the grafts is necessary to obtain adequate width and symmetry of the middle third and avoiding irregularities on the dorsum [9]. Even with proficiency, this step is often time consuming. When additional layers of cartilages are required, they might be difficult to position and fix accurately [10].

The amount of time needed for accurate placement could be a reason for a relative underuse during initial surgery: In his revision surgery experience, Daniel [11] found out as little as 5 % of patients having prior PSG 11.

In an effort to correct quickly and smoothly even small defects in the nasal vault, we used a personal technique of placement of free SPG secured with fibrin glue between septum and ULC, without thigh tunnel to support them and without suturing.

## Surgical technique

**Fig. 1:**
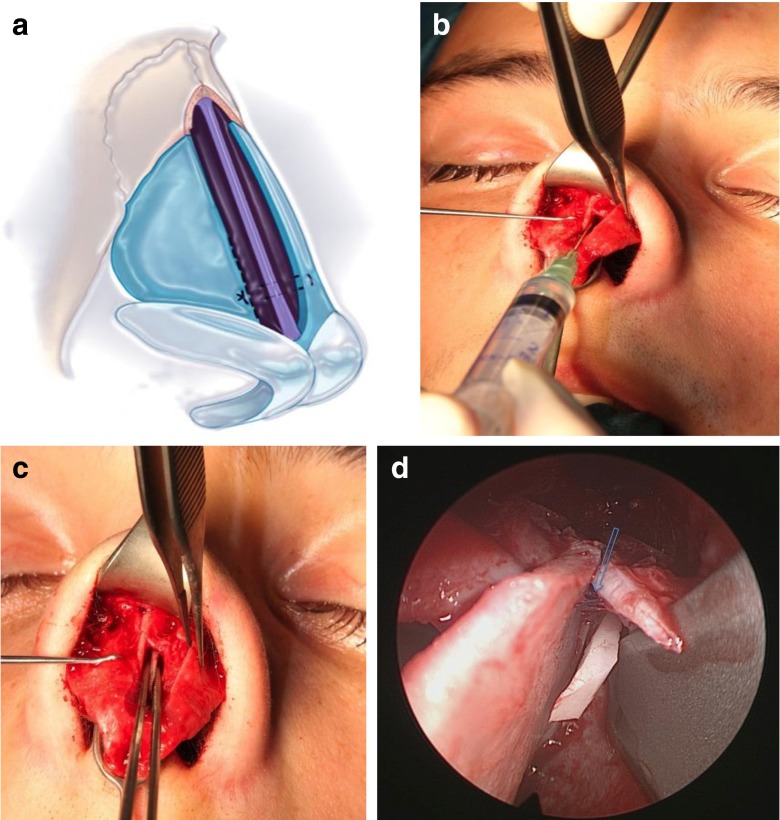
**a**, **b**, **c** Insertion of the free SPG. **a** ULC have been reattached with a U-loop suture. **b** A few drops of fibrin glue are instilled under the ULC. **c** SPG is introduced between ULC and nasal septum. **c** Endoscopic view with a 30° telescope showing initial positioning of the SPG. *Blue arrow* shows the 5–0 PDS suture

**Fig. 2:**
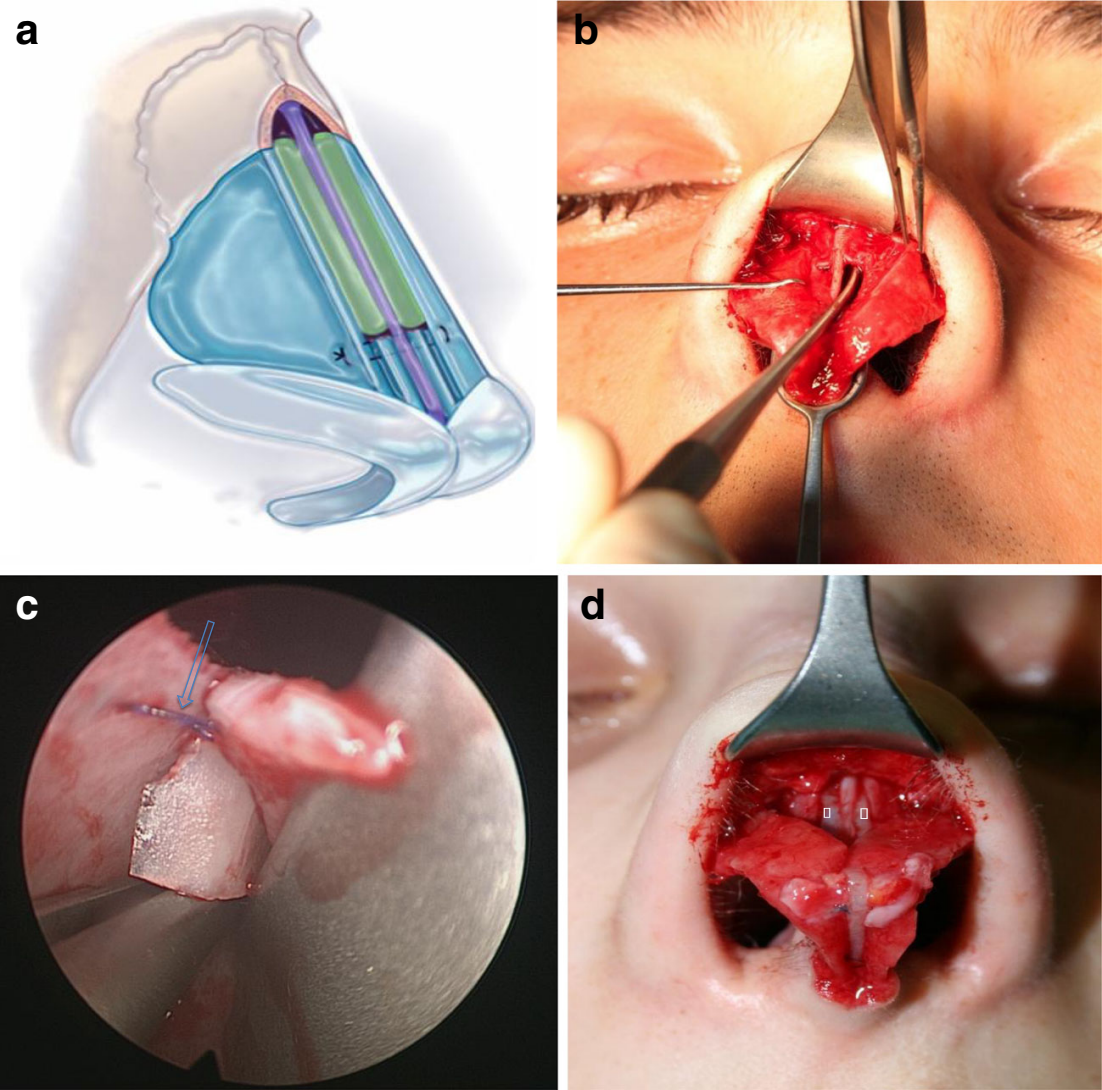
**a**,**b**,**c** Adjustment of SPG. **a** The SPG (depicted in *green*) are placed alongside the edge of nasal septum, level or a few millimetres below, depending of the amount of correction needed. **b** SPG is gently lifted with a Freer elevator. **c** Endoscopic view depicts fine adjustment of the level of the SPG under the middle vault, *blue arrow* shows ULC attachment. **d** Left and right SPG (*white dots*) in position, additional drops of fibrin glue will secure positioning

In rhinoplasty procedures, after performing modifications of the nasal cartilaginous and bony dorsum and completing osteotomies, we proceed to fixation of the ULC. They are fastened level with the nasal septum by a U-loop suture of 5–0 PDS (polydioxanone monofilament suture, Ethicon°) (Fig. 1a). Redraping of the skin permits careful visualization and palpation of the middle third. When it appears that SPG should be used, we determine the rough size of the cartilage grafts. A few drops of first component of fibrin sealant, fibrinogen (Tissucol Baxter°, Tisseel Baxter°, Baxter Healthcare Corporation, CA, USA) are applied to the cartilaginous septum (Fig. 1b). The free SPG are introduced with a forceps between the nasal septum and the ULC (Fig. 1c, d), and then gradually pushed with a Freer elevator (Fig. 2a–c), until they are slightly lower, 1 or 2 mm, than that of the dorsal septum. The skin is redraped over the nose, and again visualization and palpation evaluate adequacy of graft placement. If the graft is too large, it is removed, trimmed and replaced. In the contrary, if the amount of correction appears insufficient, the graft is gently pushed 1 or 2 mm higher. In an incremental fashion, additional pieces of cartilages are positioned laterally to the first graft if necessary. Once correction is considered adequate (Fig. 2d), the second component of fibrin sealant (thrombin) is applied to the grafts. Tip surgery is accomplished and an ultimate control is done before definitive closing of the internal or external columellar incision.

## Material and method

We performed a retrospective analysis for free SPG in 430 consecutive rhinoplasties between January 2007 and February 2011.

Postoperative follow-up extended from 1 year up to 5 years. All patients were operated by the same senior surgeon (YG). Digital photographic files were reviewed both by an experienced rhinoplastic surgeon (JD), who was not aware of the surgical technique used, and by the surgeon (YG). Face views, oblique, lateral and basal views were considered for the purpose of the study. The two senior authors (YG and JD) reached on each case a consensus as to classify the harmony of the middle third of the nose, in relationship with the bony pyramid and the tip. We classified postoperative results into four categories:

- Adequate correction of the middle third and harmony of brow tip lines
- Insufficient correction, with inverted V deformity or rigidity of brow tip lines
- Excessive width of middle third
- Asymmetry of the middle third and/or disruption of aesthetic brow tip lines.

**Fig. 3:**
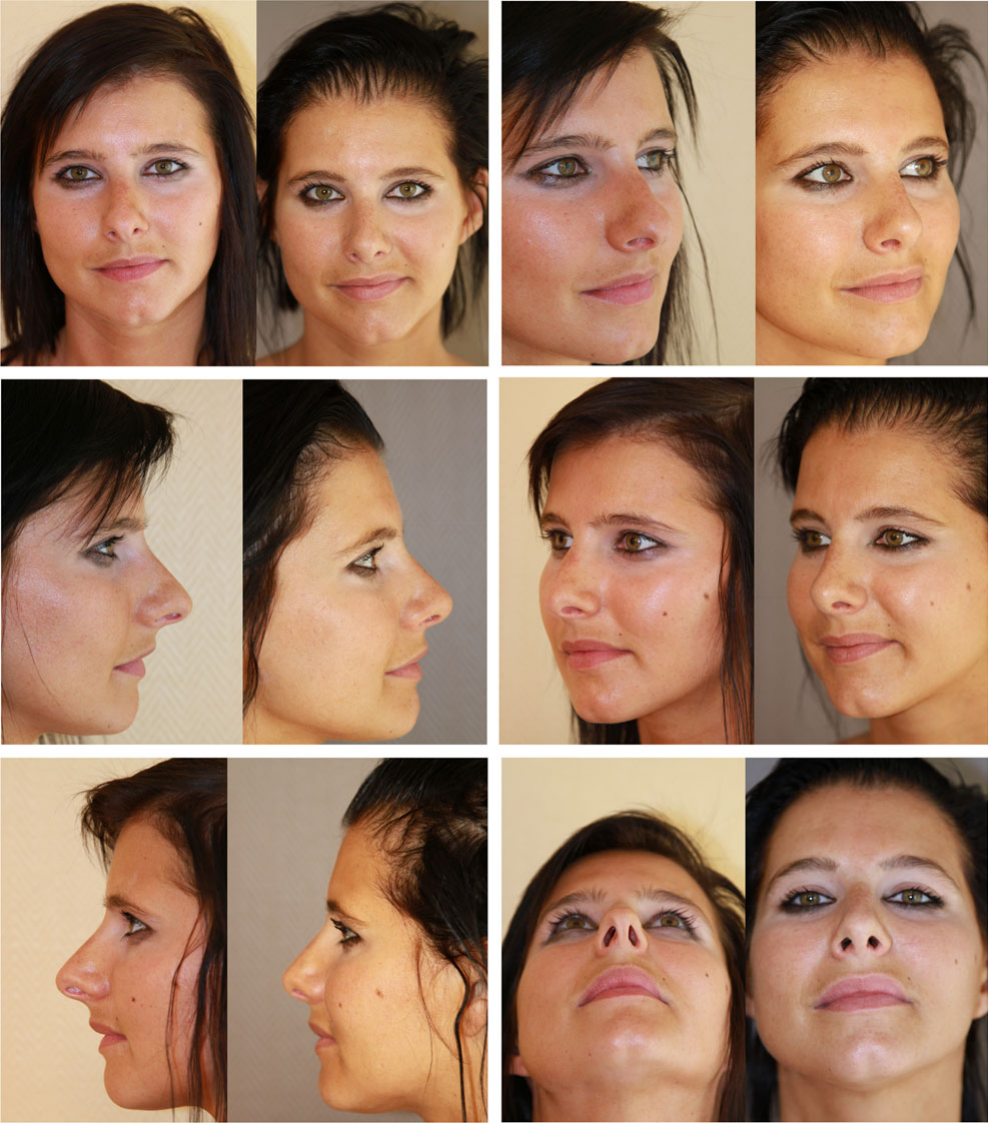
Adequate width and symmetry of middle third. Face, lateral, oblique and basal views

**Fig. 4:**
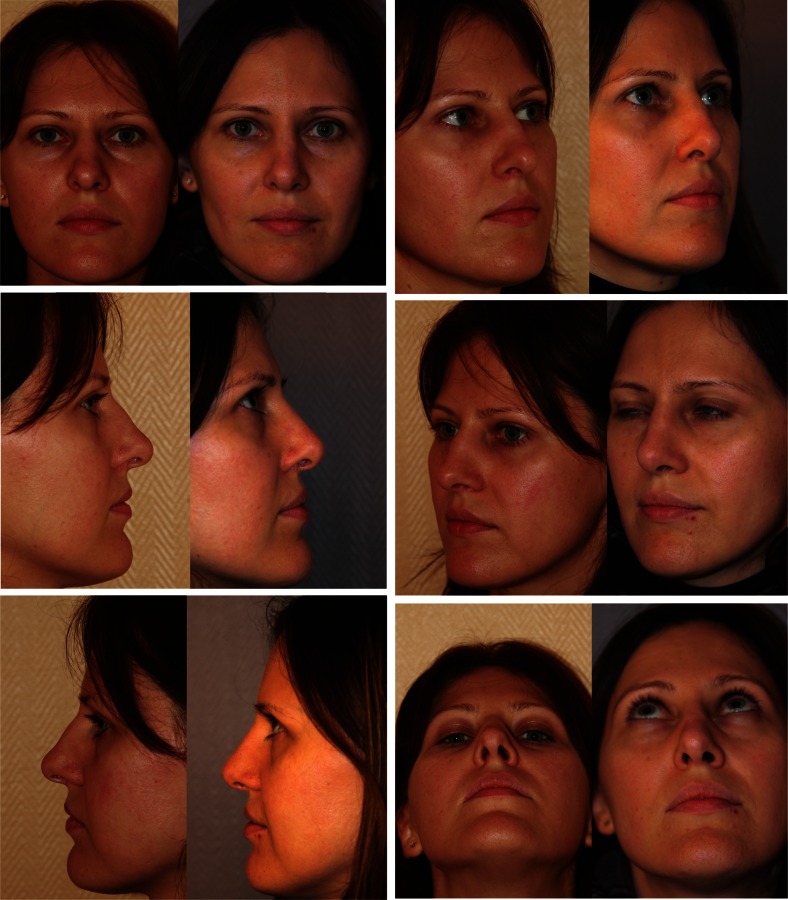
Insufficient correction of the middle third. Face, lateral, oblique and basal views

**Fig. 5:**
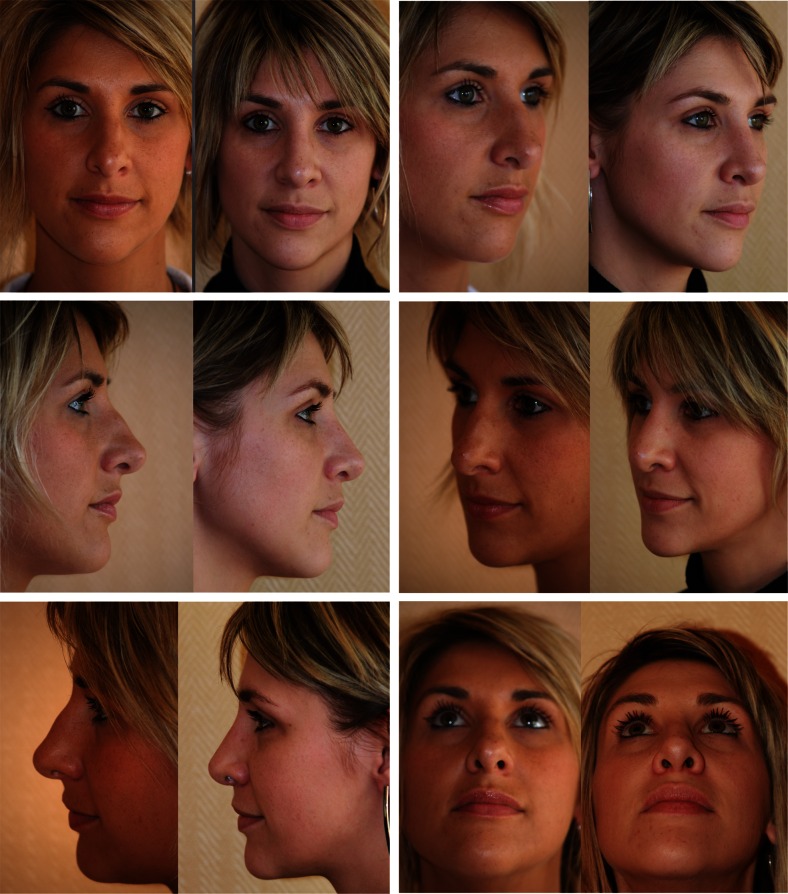
Symmetry but excessive width of middle third. Face, lateral, oblique and basal views

**Fig. 6:**
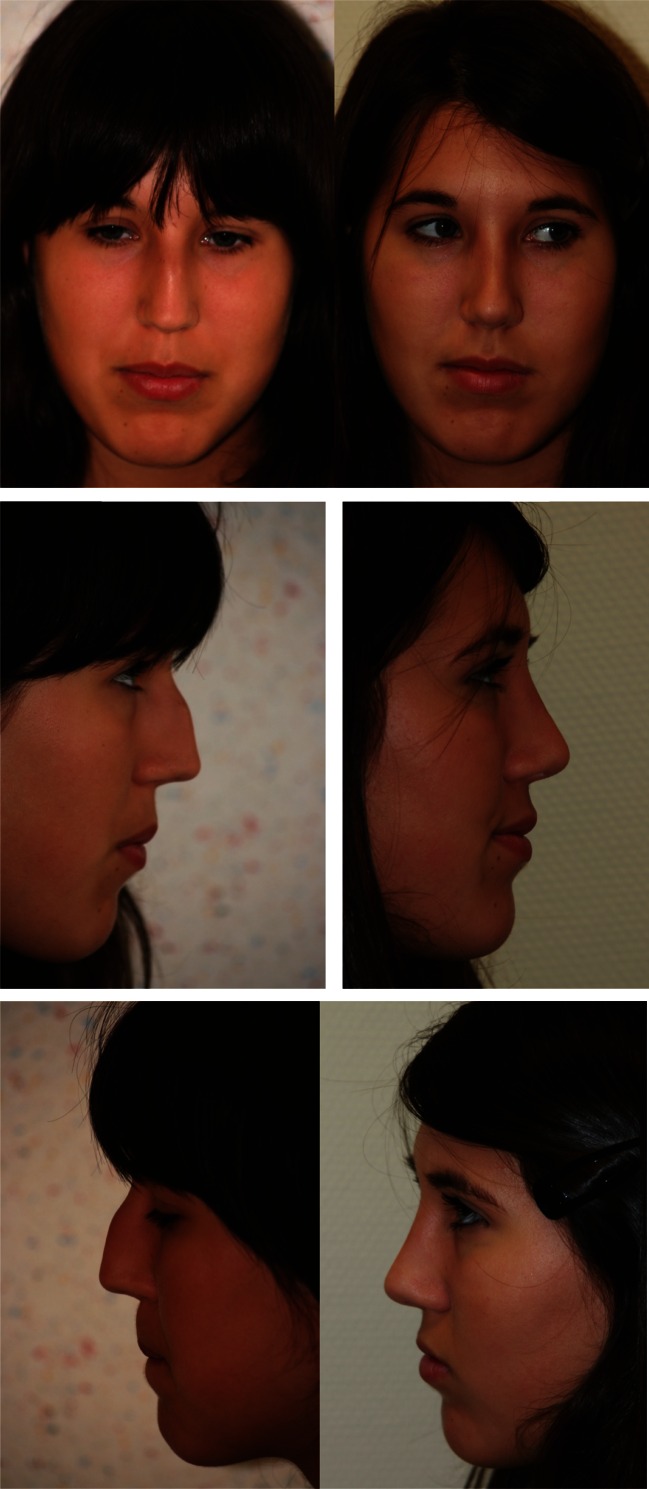
Asymmetry of middle third with collapse of the right ULC. Frontal and lateral views

Examples of our assessments are displayed in Figs. 3, 4, 5 and 6.

## Results

Overall, 420 patients were eligible for evaluation as ten patients were lost for follow-up. Surgery was performed for cosmetic or both cosmetic and functional purposes in 367 patients (87 %). Three hundred seventy-eight patients were primary cases and 42 patients (10 %) were revision cases (Table 1). SPG were placed in 251 patients (62 %) (Table 2). Free SPG were used in 218 patients (54 % of rhinoplasty patients) while classic, sutured SPG were used in 33 patients (8 %). Among the patients with free SPG asymmetric sizing of the graft was used 35 patients (16 % of grafts placed) while multilayered grafts were used in 19 patients (9 % of grafts placed). External rhinoplasty was the most common surgical route used in 402 patients while endonasal approach accounted for 18 patients.

**Table 1:** Patient’s population

Number of eligible patients	420	Type of procedure
Primary procedures	196	Aesthetic
*n* = 378	131	Aesthetic and functional
	51	Functional
Revision procedures	31	Aesthetic
*n* = 42	9	Aesthetic and functional
	2	Functional

**Table 2:** Type of spreader grafts used

Number of open rhinoplasty patients	*n* = 402	%
All spreader grafts included	251	62 %
Free SPG	218	54 %
Subtypes of SPG	Asymmetric grafts n = 35	16 % of free SPG
	Multilayered grafts n = 19	9 % of free SPG
Sutured SPG	33	8 %

Asymmetric grafts means that the size of the graft were not equal on each side

Multilayered grafts means that more than one piece of cartilage were used to obtain adequate correction of the middle vault

Among the 169 patients where no SPG were placed, 71 % of good control of middle third was noted, while 4 % of the patients presented a too thin middle third, 5 % too wide and 21 % with asymmetry of the middle third (Table 3).

**Table 3:** Aesthetic results—no spreader grafts used

No SPG	169 patients without SPG	
Middle third-aesthetic brow tip lines		
Symmetric adequate	120	71 %
Symmetric thin	6	4 %
Symmetric wide	8	5 %
Asymmetric disruption	35	21 %

In the group of 33 patients with sutured SPG adequate middle third was noted in 70 %, too thin in 3 %, too wide in 9 % and asymmetry or disruption of aesthetic lines in 18 % of the patients (Table 4).

**Table 4:** Aesthetic results—conventional sutured spreader grafts

Fixed SPG	33 patients with SPG	
Middle third-aesthetic brow tip lines		
Symmetric adequate	23	70 %
Symmetric thin	1	3 %
Symmetric wide	3	9 %
Asymmetric disruption	6	18 %

In the group of 218 patients with free SPG (Table 5), postoperative evaluation by the authors showed that middle third width and aesthetic brow nasal lines were considered as symmetric and adequate in 168 patients (77 % of the grafts), symmetric but too thin in six patients (3 %), symmetric but too wide in six patients (3 %). Asymmetry of the middle third of the nose or disruption of the aesthetic lines of the nose was observed in 38 patients (17 %).

**Table 5:** Aesthetic results—free spreader grafts

Fixed SPG	218 patients with SPG	
Middle third-aesthetic brow tip lines		
Symmetric adequate	168	77 % of the grafts
Symmetric thin	6	3 % of the grafts
Symmetric wide	6	3 % of the grafts
Asymmetric disruption	38	17 % of the grafts

We performed a statistical evaluation within the three groups: no SPG, fixed SPG and free SPG with a Statgraphics Centurion° software. Since *P* value is largely greater than that of statistical significance at 0.95, there is no statistically significance amongst the three samples tested.

No signs of early displacement of the graft were clinically observed. No graft extrusion towards the dorsum was observed. One patient with revision rhinoplasty (including dorsal augmentation with conchal cartilage and fascia temporalis grafting) presented an infection with drainage through the dorsal skin but it does not appeared to be specifically related to the free SPG technique.

## Discussion

Fixation of SPG is an issue in rhinoplasty. While Sheen [1, 2] and Constantian [12] described initially the placement into a submucous tunnel, suturing techniques have been reported by various authors. Johnson [3] described loop sutures placed between SPG and saddled over the septum. Suturing techniques really emerged with the advent of open rhinoplasty procedures [13].

In our technique, free SPG were used as a customizable material between the ULC and septum. Overall, the whole process of tailoring the free SPG and placing it took only a few minutes. It allowed us to correct minor defect or asymmetries in the middle third, and the versatility of the placement increased the frequency of use of SPG placement. As free SPG are placed cephalically to the fixation of ULC to the septum, they reduce the risk of creating excessive bulk and inadequate fullness in the supratip area. Positioning through an endonasal approach was also readily achievable.

In functional rhinoplasty, free SPG provide less opening of the nasal valve than a SPG fixed along the dorsal edge and running to the anterior septal angle. We did not intend to correct marked deviation of the dorsum with those grafts.

**Fig. 7:**
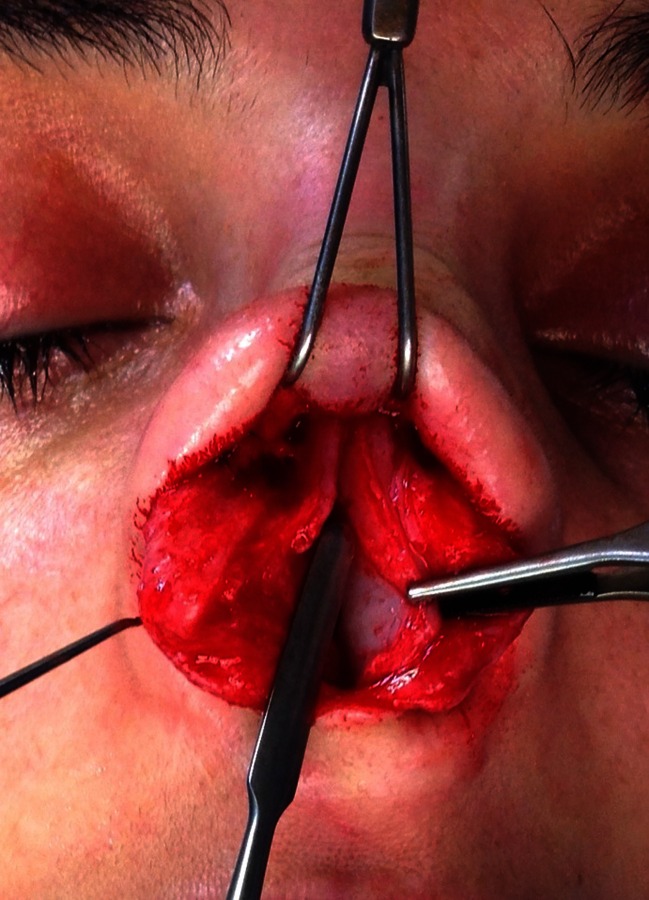
Operative view during revision surgery at 2 years. Free SPG is found alongside the dorsal septum after liberation of ULC

We could not detect sign of early displacement of the graft. On occasion during revision surgery, we found that SPG were in place and underwent no specific resorption, as depicted in Fig. 7.

No specific complications were related to the use of fibrin glue. This is in contrast to a study of André [14] who described in functional rhinoplasty patients the opening the nasal valve with SPG without dividing the ULC and securing them with 2-cyano-butyl-acrylate glue. It resulted in an unacceptable rate of postoperative infection (three patients out of eight patients reported).

Fibrin sealants have been used widely in Europe for 15 years. Concerns exist regarding the use of blood derivates (fibrin glue contains human fibrinogen, human thrombin and bovine aprotinin), but the European’s Agency Committee for Medical Products for Human Use concluded that the use of these products is safe [15].

Additional costs are associated with fibrin glue use. The cost is limited as an amount of 1 ml of fibrin glue per patient is sufficient. In Europe, the price in 2014 was for 1 ml Tissucol° or Tisseel° (Baxter°, Baxter Healthcare Corporation, CA, USA) 104 € or approximately US$120. Regarding the total costs involved in surgery, anesthesia, medications and hospital stay, the additional charge is regarded as acceptable. Nevertheless, the patient should be informed that fibrin glue, containing human and bovine blood derivates, would be possibly used during surgery.

The evaluation of the results of SPG is difficult and subjective. Few objective quantitative assessments have been described [16]. We estimated that the best way to assess appropriateness of SPG was to appreciate width and symmetry of the middle nasal vault, as well as the brow tip aesthetic lines referred by Tardy [17] and Sheen [18]. The pertinence of the method of assessment could be debated, as several factors influence these issues. For example, brow tip lines are determined by adequate harmony between nasal bones and middle third, and are affected not only by the width of middle third, but also by overly narrow or wide osteotomies or persisting deviation of nasal pyramid. This was the reason for evaluating the results by an independent and experimented observer reviewing patient’s pictures and by the surgeon.

No statistical differences were detected between the different groups, regardless of the technique used. This fact does not imply that SPG were of no use, but that statistical analysis was hindered by a bias: the more severe the deformity, the more the surgeon is prone to use strong and fixed reconstructive material to achieve structural correction. The deviated nose, the short nose, the revision surgery or the posttraumatic nose are much more likely to need fixed SPG. It shows interestingly that although surgical techniques are individually tailored, we are left over time with a comparable proportion of patients with unsatisfactory correction. This may be explained by misjudgment during surgery or unforeseen evolution during the healing process. For this reason, the purpose of the study was not to assess the superiority of a technique over another but to evaluate if our free SPG technique leads to an acceptable rate of correction of the middle third. The high percentage of free SPG compared to conventional SPG in our patients is probably related to the indications for surgery, with a majority of cosmetic indications for surgery.

The major advantage was the user-friendliness and versatility of free SPG, accounting for a relatively high rate of use (62 % of rhinoplasty patients at the time of the study), and a much higher rate in our recent surgeries. Moreover, certain authors advocate bilateral SPG each time that the middle vault has been opened during rhinoplasty [9]. This simplified method could help preventing the underuse of conventional SPG often reported by authors [9, 11].

## Conclusion

Free SPG placement during a rhinoplasty procedure when limited amount of support was needed appeared easy and timesaving. The placement in an incremental fashion as suggested by visualization and palpation proved a very flexible maneuver and allows instant adjustability and fine trimming. Over time, it does not appear that unexpected displacement of the grafts occurred in early postoperative period. At long term, we observed occasionally during revision surgeries that the free SPG were integrated in suitable position. The evaluation of cosmetic results by an independent observer and the surgeon find out an acceptable rate of adequate correction to the middle third of the nose. Free SPG behave as a filler material, easy to adjust, to correct minor to moderate irregularities or to prevent middle third collapse. The ease of placement might help preventing underuse of SPG often detected in revision surgeries. Over the last 8 years, the author’s modification of technique has dramatically changed our approach to SPG and increased considerably both frequency of use and straightforwardness of placement.
